# Synchronous Nasopharyngeal Carcinoma and Rectal Adenocarcinoma Treated With Integrated Multimodal Therapy: A Case Report and Literature Review

**DOI:** 10.7759/cureus.113011

**Published:** 2026-07-20

**Authors:** Najd S AlGazlan, Ali AlFakeeh, Mohammed AlDohan, Ali M AlZahrani

**Affiliations:** 1 Medical Oncology Department, Comprehensive Cancer Center, King Fahad Medical City, Riyadh, SAU; 2 Radiation Oncology Department, Comprehensive Cancer Center, King Fahad Medical City, Riyadh, SAU

**Keywords:** concurrent chemoradiotherapy, double primaries, immunotherapy, nasopharyngeal carcinoma, pembrolizumab, rectal cancer, short-course radiotherapy

## Abstract

Multiple primary malignancies are rare events, but when they happen, especially for curable cancers, they can cause management challenges. Locally advanced nasopharyngeal carcinoma is currently treated with induction chemotherapy followed by concurrent chemoradiotherapy, while locally advanced rectal cancer is currently treated with total neoadjuvant therapy, including chemotherapy and radiotherapy, followed by surgery. To integrate both treatments is really challenging and requires meticulous planning and monitoring to have a successful outcome with manageable toxicity.

We present the case of a synchronous locally advanced nasopharyngeal carcinoma and locally advanced rectal adenocarcinoma in a 63-year-old Saudi Arabian man who was treated with induction chemoimmunotherapy including capecitabine and oxaliplatin (CAPEOX) with pembrolizumab for four cycles, starting in November 2023. This was followed by short-course radiotherapy to the pelvis, two additional cycles of CAPEOX plus pembrolizumab, definitive concurrent chemoradiotherapy to the nasopharynx, and finally abdominoperineal resection of the rectal tumor. He tolerated the procedure very well, and he is still in complete remission till his last follow-up in April 2026.

The present report may possibly be the first to report a case of synchronous locally advanced nasopharyngeal carcinoma and locally advanced rectal adenocarcinoma that has been treated successfully by integrating immunotherapy into the standard treatment for both.

## Introduction

Multiple primary malignancies are characterized by the presence of two or more histologically distinct primary tumors occurring in the same individual, which are not metastatic deposits or recurrences of a previous malignancy. Multiple primary malignancies are generally categorized into synchronous tumors, which are diagnosed simultaneously or within a six-month period, and metachronous tumors, which are identified sequentially after six months, indicating different biological behaviors and clinical consequences [1]. The reported incidence of multiple primary malignancies varies from approximately 0.7% to over 10%, with this variability attributed to differences in population demographics, cancer surveillance methods, and advancements in diagnostic technologies [2]. Synchronous multiple primary malignancies, which appear in literature in less than 2%, pose distinct diagnostic and therapeutic challenges, especially when both malignancies necessitate curative-intent, multimodal treatment approaches that may overlap or conflict in terms of timing, toxicity, and sequencing [3]. Management typically requires a highly personalized, multidisciplinary strategy to balance treatment effectiveness against cumulative toxicity and patient tolerance [4]. Additionally, the lack of standardized guidelines for rare tumor combinations complicates clinical decision-making and highlights the necessity of case-based evidence [5]. Nasopharyngeal carcinoma is a specific epithelial malignancy that originates from the nasopharyngeal mucosa, exhibiting a unique geographic distribution and a strong etiological link to Epstein-Barr virus infection, particularly in endemic areas [6]. Nasopharyngeal carcinoma is noted for its high sensitivity to radiation and chemotherapy, establishing radiotherapy as the fundamental treatment method [7].

In cases of locoregionally advanced disease, concurrent chemoradiotherapy using platinum-based regimens has been recognized as the standard care, showing considerable enhancements in overall survival and locoregional control compared to radiotherapy alone [8]. Recently, the introduction of immune checkpoint inhibitors that target programmed death-1 (PD-1) has significantly altered the treatment landscape for recurrent or metastatic nasopharyngeal carcinoma [9-12]. Rectal adenocarcinoma, a significant subtype of colorectal cancer, is generally treated using a multimodal strategy that combines radiotherapy, systemic chemotherapy, and surgical resection to achieve the best oncological results [13]. Neoadjuvant treatment methods have progressed notably, with total neoadjuvant therapy becoming a favored strategy for locally advanced disease to facilitate tumor downstaging and enhance systemic control [14]. Short-course (25 Gy in five days) and long-course radiotherapy (45-50.4 Gy over 5-6 weeks) and systemic chemotherapy and delayed surgical intervention have been substantiated in randomized trials in showcasing enhanced disease-related outcomes and improved adherence to systemic therapy [15,16].

In this report, we present a distinctive case of synchronous locally advanced nasopharyngeal carcinoma and rectal adenocarcinoma that was effectively managed through a customized multidisciplinary approach, which included chemoimmunotherapy, short-course radiotherapy for rectal cancer, and definitive concurrent chemoradiotherapy targeting the nasopharynx and bilateral neck lymphatics, followed by abdominoperineal resection. This case underscores the viability and efficacy of a personalized, evidence-based strategy for addressing complex synchronous malignancies while optimizing both local and systemic disease management.

## Case presentation

Our patient was a 63-year-old Saudi Arabian man who was a nonsmoker and does not drink alcohol. He has no chronic medical illnesses, has no family history of malignancy, and was fully active with an Eastern Cooperative Oncology Group (ECOG) Performance Status of 1. In October 2023, he noticed fresh blood in his stools associated with pain. During further inquiries, he reported a persistent feeling of incomplete evacuation and a change in bowel habits. He also reported epistaxis on different occasions that became more consistent recently. He was evaluated at his regional hospital by a nasal scope that showed a mass at the right side of his nasopharynx and a colonoscopy that showed a fungated ulcerated rectal mass at around 10 cm from the anal verge, and biopsies were obtained from both. Histopathology of the nasopharyngeal mass came as Epstein-Barr virus-positive non-keratinizing nasopharyngeal carcinoma, while the rectal mass was reported as invasive moderately differentiated adenocarcinoma, mismatch repair-proficient.

**Figure 1:**
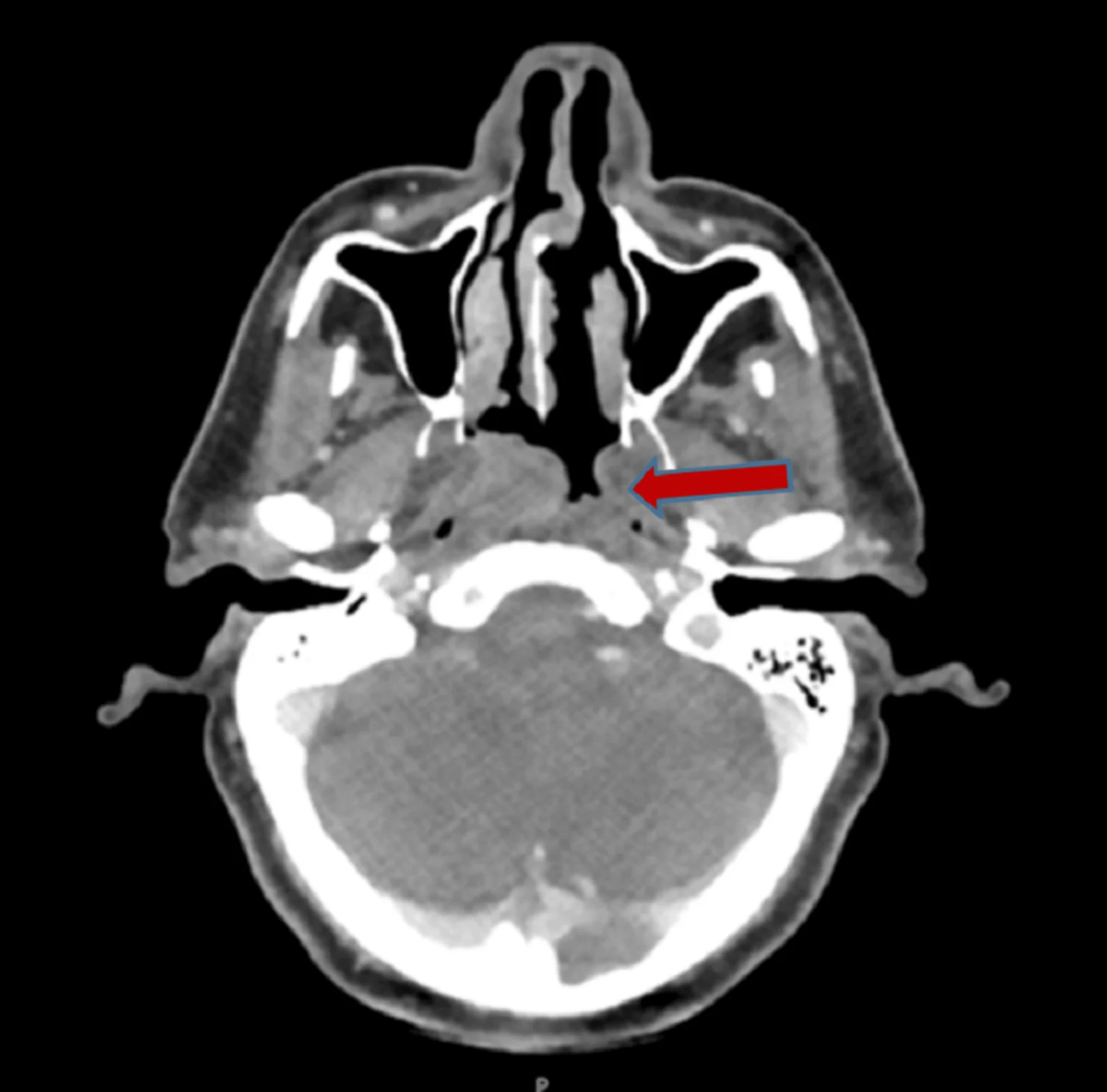
Computed tomography of the nasopharynx before treatment (arrow)

**Figure 2:**
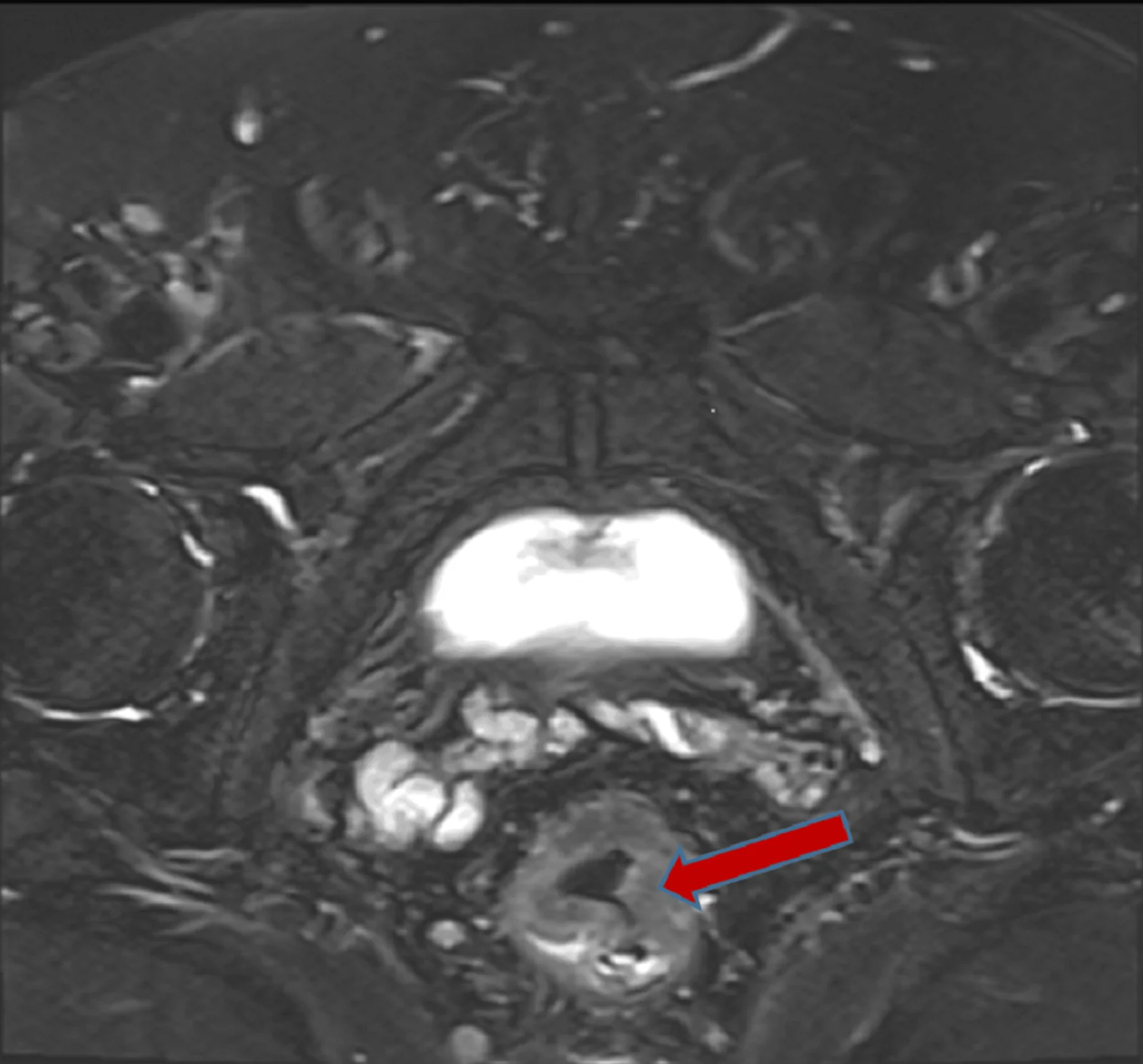
Magnetic resonance imaging of the pelvis showing the rectal tumor before treatment (arrow)

**Figure 3:**
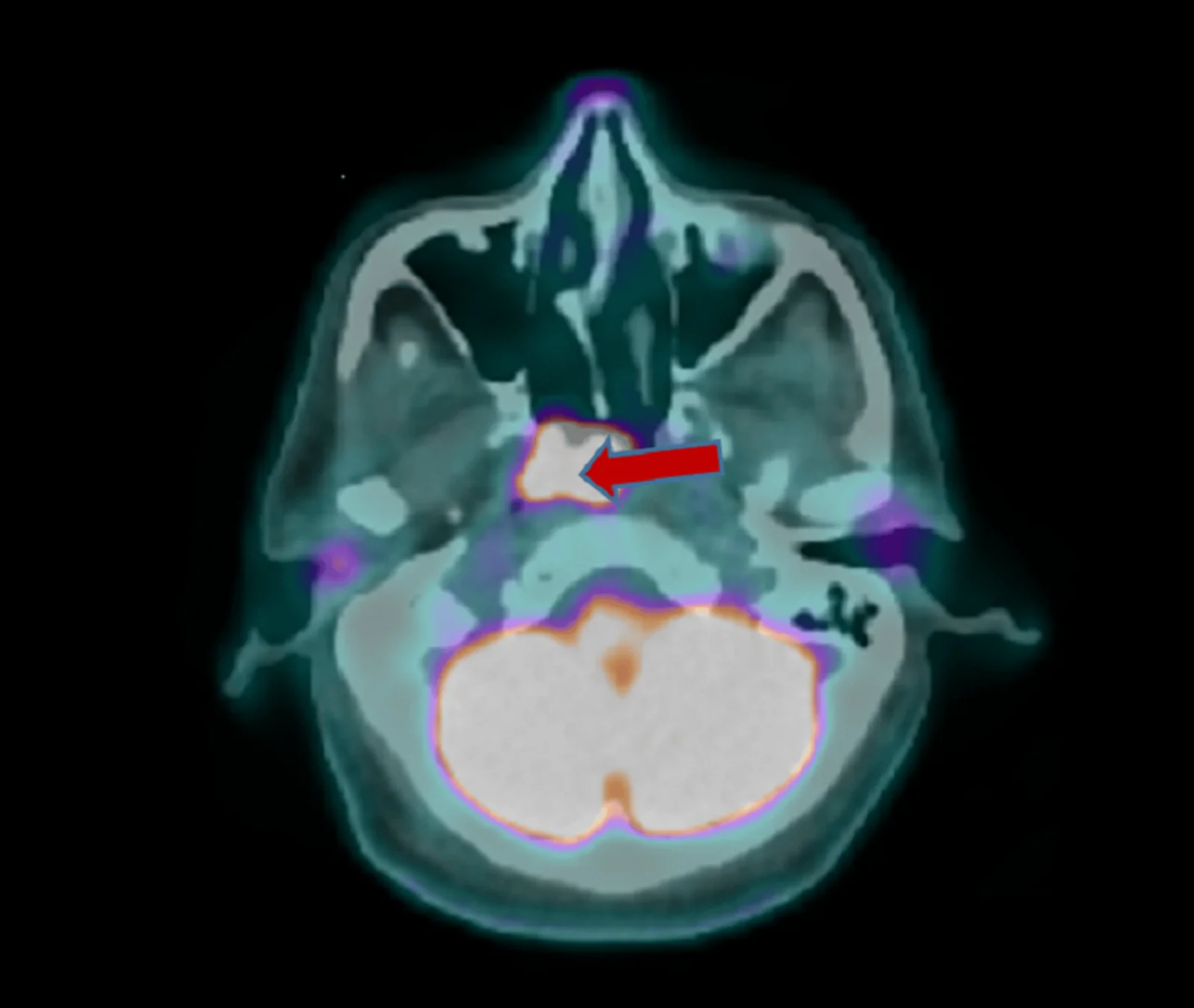
Positron emission tomography computed tomography showing the nasopharyngeal tumor before treatment (arrow)

**Figure 4:**
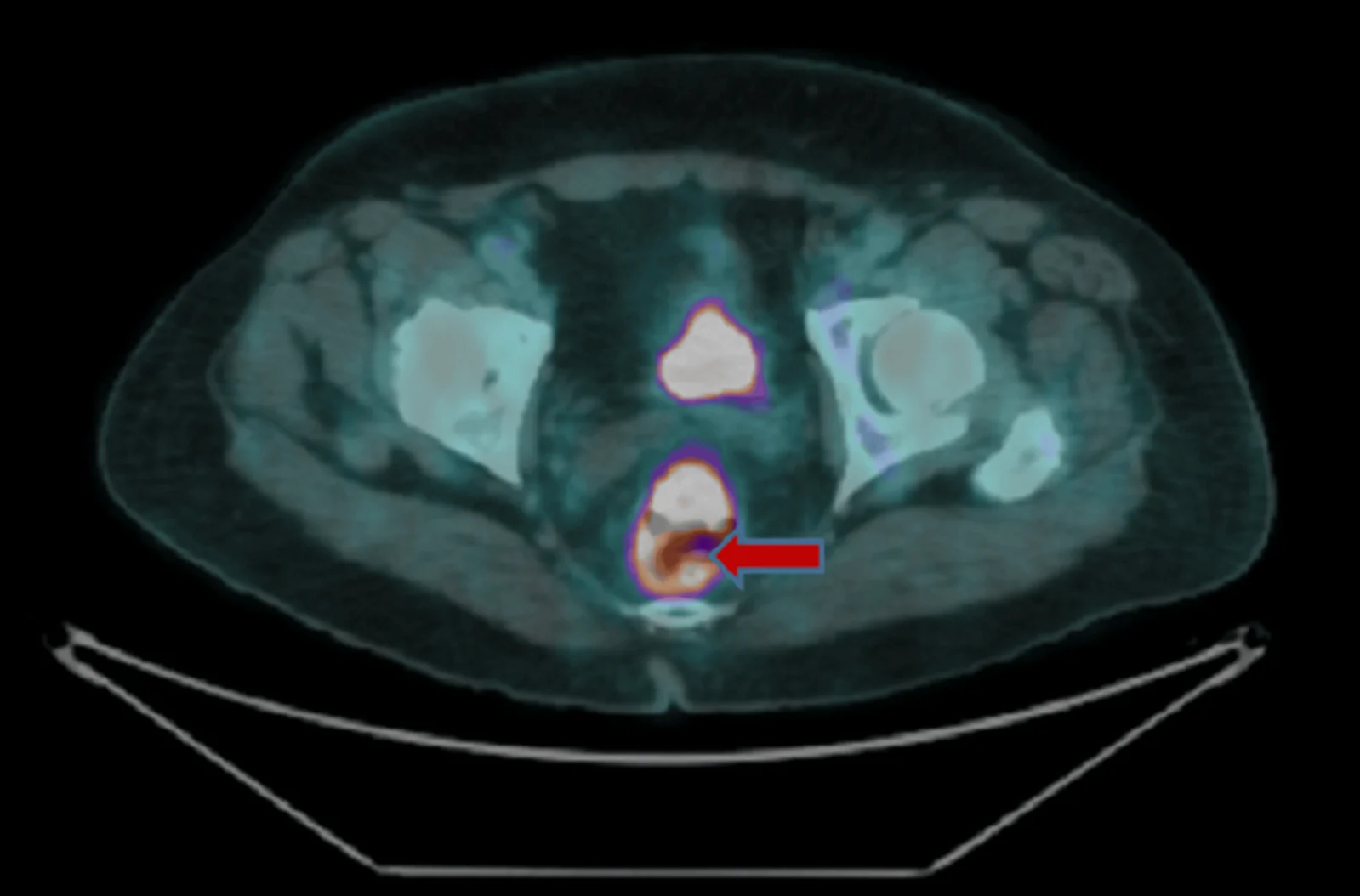
Positron emission tomography computed tomography showing the rectal tumor before treatment (arrow)

Full staging workup was carried out including computed tomography (CT) of the head and neck (Figure 1) and magnetic resonance imaging (MRI) of the head and neck, CT of the chest, abdomen, and pelvis, and MRI of the pelvis (Figure 2). He also had whole-body positron emission tomography (PET) CT (Figures 3-4). He was found to have stage III (cT4N1M0) rectal adenocarcinoma and stage IV-A (cT1N3M0) nasopharyngeal carcinoma according to the American Joint Committee on Cancer (AJCC) tumor-node-metastasis (TNM) staging system [17]. The case was discussed extensively at the tumor board multidisciplinary meeting, and it was decided to start chemotherapy that works well with rectal cancer and can cover nasopharyngeal carcinoma and also to add immunotherapy that can augment the effect on nasopharyngeal carcinoma. It was also decided to give short-course radiotherapy for rectal cancer so we will not delay the definitive treatment for nasopharyngeal carcinoma. The surgical part of the rectum was set at the end.

**Figure 5:**
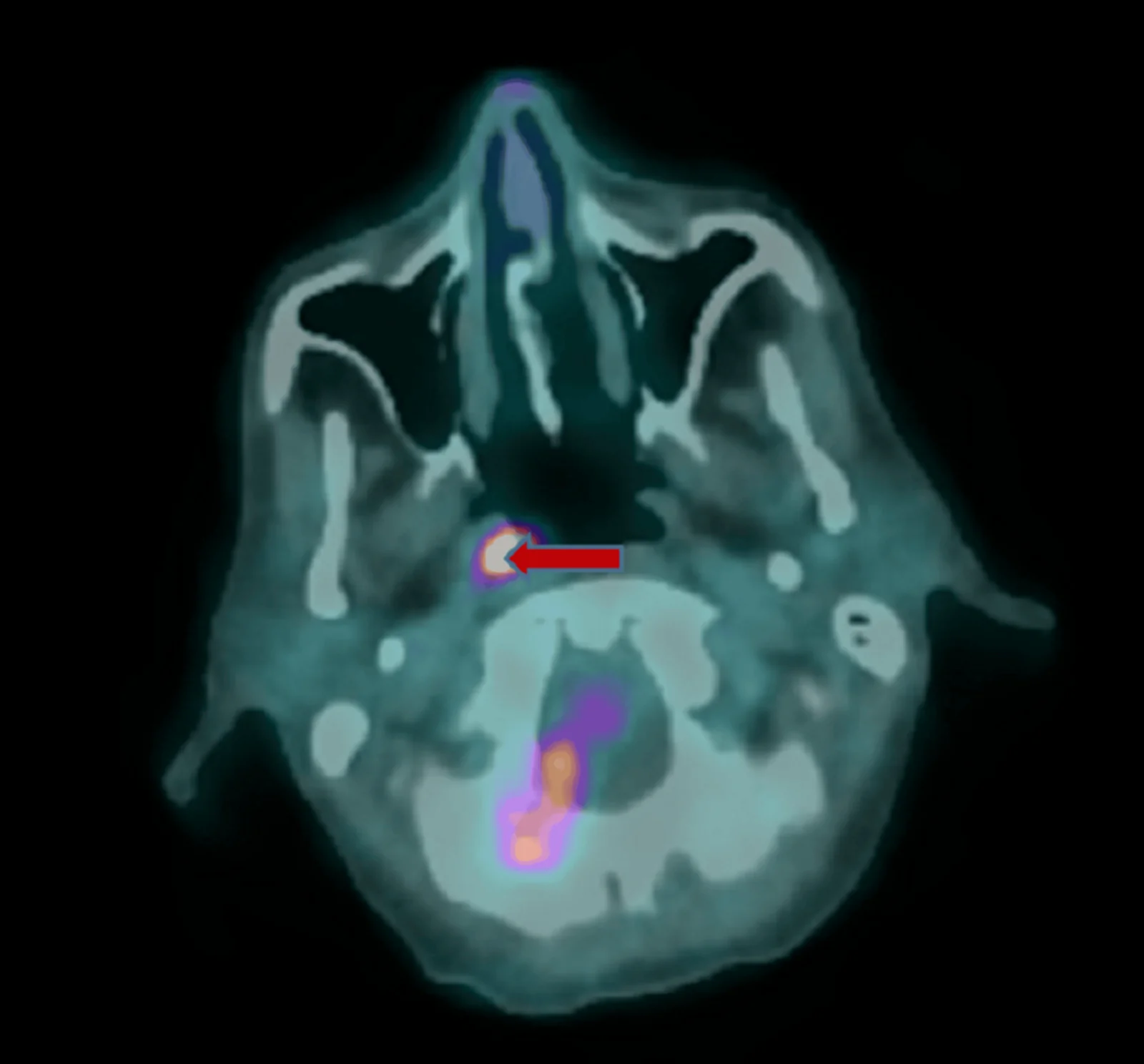
Positron emission tomography computed tomography of the nasopharynx showing good response after induction treatment (arrow)

**Figure 6:**
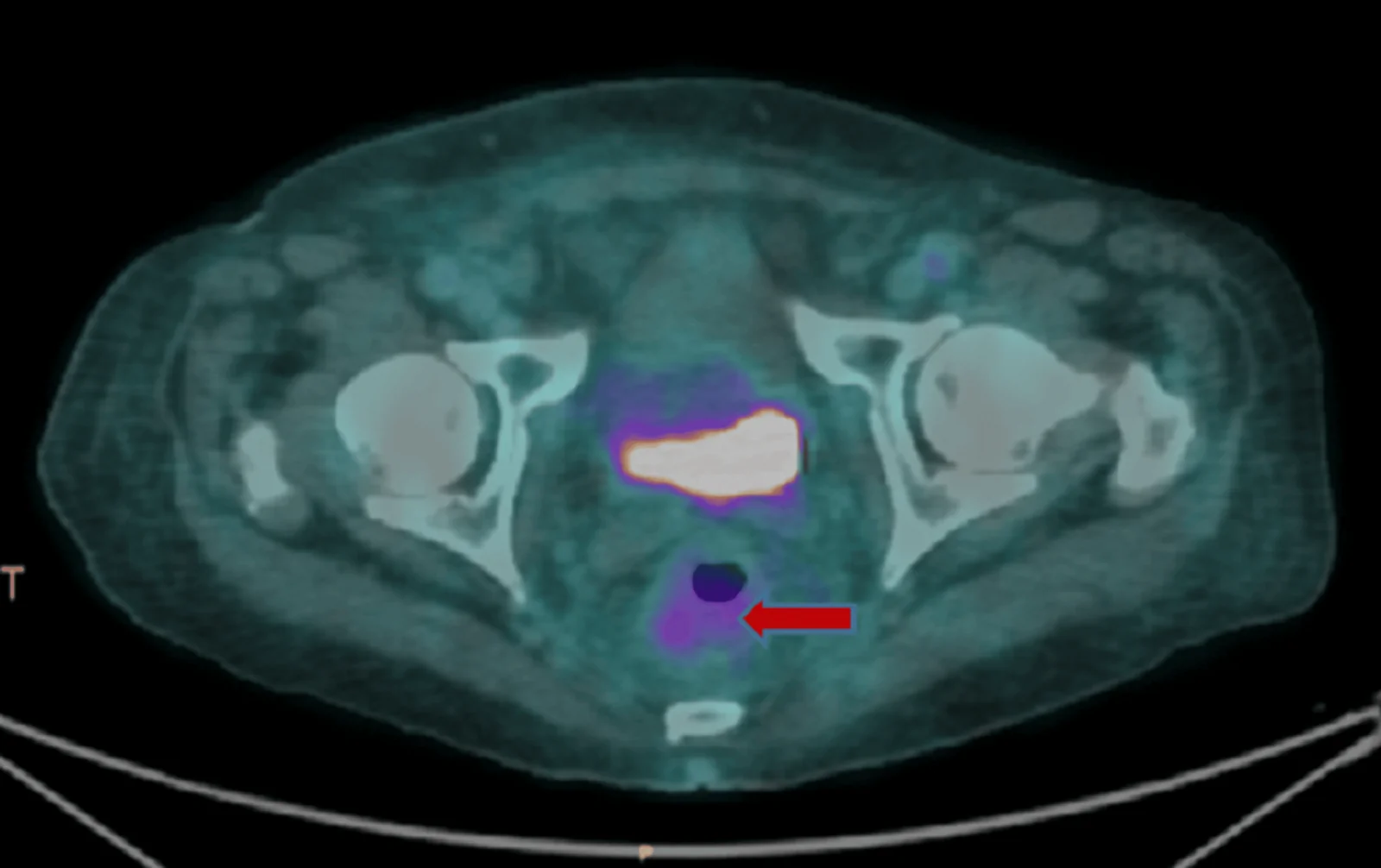
Positron emission tomography computed tomography of the pelvis showing good response of rectal tumor after induction treatment (arrow)

The patient began treatment (month 0) with four cycles of capecitabine 1000 mg/m² orally twice daily for 14 days and oxaliplatin 130 mg/m² intravenously (CAPEOX) in conjunction with pembrolizumab 200 mg intravenously every three weeks, which ended in month 3, with a good partial response for both primaries as shown on PET CT (Figures 5-6). Following this, he underwent short-course radiotherapy (5×5 Gy) targeting the rectum, which was completed in month 4. Afterward, two more cycles of CAPEOX combined with pembrolizumab were administered, which concluded in month 5. Definitive concurrent chemoradiotherapy with weekly cisplatin 40 mg/m² intravenously was subsequently provided to the nasopharynx and bilateral cervical lymph nodes, delivering a total dose of 70 Gy over 33 fractions, which was completed in month 8. Surgical intervention for the rectal tumor was delayed because the patient developed acute intestinal obstruction due to an umbilical hernia, but he underwent a laparoscopic abdominoperineal resection of the rectum and anus in month 12. The choice to conduct an abdominoperineal resection instead of an anterior resection was determined during the surgical procedure. The colorectal surgeon discovered significant pelvic fibrosis, which complicated the possibility of sphincter-preserving surgery and increased the risk of complications. Consequently, abdominoperineal resection was deemed the safer alternative, likely to yield a more favorable long-term functional outcome and enhance the patient's quality of life. Prior to the operation, the patient was informed and gave consent for both anterior resection and abdominoperineal resection, enabling the surgical method to be selected based on the findings observed during the procedure. Histopathology was pT3N1 with extensive treatment-related necrosis and tumor regression grade (TRG) 1 according to the AJCC/College of American Pathologists (CAP) tumor regression grading system.

## Discussion

Synchronous multiple primary malignancies are uncommon, with reported incidence ranging from 0.002% to 1.96% [3]. The coexistence of nasopharyngeal carcinoma and rectal adenocarcinoma is exceptionally rare and, to our knowledge, has not been previously described in the literature. Published reports indicate that synchronous nasopharyngeal carcinoma most often occurs with other head and neck malignancies, particularly oropharyngeal squamous cell carcinoma, where shared viral oncogenic mechanisms such as Epstein-Barr virus and human papillomavirus may play a role [18]. Less frequently, nasopharyngeal carcinoma has been reported in association with distant solid tumors, including breast carcinoma, gastric adenocarcinoma, and thyroid malignancies [19-21]. These reports are limited to isolated cases and do not demonstrate a consistent biological or clinical pattern.

The absence of reported cases involving colorectal malignancies likely reflects the distinct etiological pathways underlying these diseases. Nasopharyngeal carcinoma is strongly associated with Epstein-Barr virus and has a unique geographic distribution, whereas rectal adenocarcinoma is primarily linked to chromosomal instability, environmental factors, and lifestyle-related risks [22]. The lack of shared risk factors may explain the rarity of this synchronous presentation. Management of synchronous malignancies is not standardized and requires an individualized, multidisciplinary approach. The selection of capecitabine and oxaliplatin (CAPOX) as the backbone of systemic therapy in this case warrants careful consideration. While CAPOX is not a standard regimen for nasopharyngeal carcinoma, its use was based on the need for a unified systemic approach capable of addressing both malignancies simultaneously. Capecitabine, an oral fluoropyrimidine, has demonstrated clinically meaningful activity in nasopharyngeal carcinoma in both metastatic and adjuvant settings, including improved survival outcomes when used as maintenance therapy following chemoradiotherapy [23]. In addition, capecitabine has been explored as a substitute for infusional 5-fluorouracil in combination regimens with cisplatin, with acceptable efficacy and tolerability [24].

In contrast, oxaliplatin does not have an established role in nasopharyngeal carcinoma, which is traditionally considered a cisplatin-sensitive disease. However, oxaliplatin has demonstrated broad antitumor activity across gastrointestinal malignancies and offers a non-cross-resistant platinum alternative. In the context of synchronous malignancies, its inclusion was intended to maintain optimal efficacy against rectal adenocarcinoma while providing cytotoxic synergy with capecitabine. Importantly, the use of CAPOX allowed for effective systemic disease control without compromising the ability to deliver subsequent cisplatin-based concurrent chemoradiotherapy, which remains the standard of care for nasopharyngeal carcinoma. This sequencing minimized overlapping toxicities and preserved treatment intensity for curative-intent therapy. Although CAPOX is not validated in nasopharyngeal carcinoma, its use in this context represents a pragmatic and biologically plausible approach tailored to the challenges of synchronous malignancies. The favorable clinical and pathological response observed in this case supports the feasibility of such an approach and highlights the need for further investigation.

The addition of pembrolizumab further strengthened this strategy, given the well-established immunogenicity of Epstein-Barr virus-associated nasopharyngeal carcinoma and the emerging role of immune checkpoint inhibitors in its management. Pembrolizumab was chosen as a representative anti-PD-1 agent due to the proven effectiveness of other PD-1 inhibitors such as sintilimab, toripalimab, and camrelizumab in treating locally advanced nasopharyngeal carcinoma, indicating a class effect associated with PD-1 blockade. There are a few trials that showed benefit for adding immunotherapy for the treatment of locally advanced nasopharyngeal carcinoma. The CONTINUUM trial, a multicenter phase III trial, evaluated the "full-course" integration of the PD-1 inhibitor sintilimab in 425 patients with high-risk locoregionally advanced nasopharyngeal carcinoma. The three-year event-free survival was significantly higher in the sintilimab group (86% vs. 76%), representing a 41% reduction in the risk of recurrence or death [25]. The DIAMOND trial, a phase III non-inferiority trial, investigated a cisplatin-sparing strategy by integrating the PD-1 inhibitor toripalimab. The study successfully met its non-inferiority endpoint, with a three-year failure-free survival of 88.3% in the experimental arm versus 87.6% in the standard arm [26]. A randomized phase II trial evaluated a "sandwich" approach using toripalimab specifically in patients with high pretreatment Epstein-Barr virus DNA levels. The protocol involved two cycles of neoadjuvant toripalimab before concurrent chemoradiotherapy, followed by maintenance adjuvant toripalimab. At a median follow-up of 37.8 months, the two-year progression-free survival was 92% in the toripalimab arm compared to 74% in the placebo arm [27]. All these trials indicated that anti-PD-1 has a significant impact on the outcome of locally advanced nasopharyngeal carcinoma and support our approach of adding pembrolizumab, which is another anti-PD-1, to the treatment protocol.

For the rectal adenocarcinoma, a short course of radiotherapy with systemic chemotherapy was chosen as part of a comprehensive neoadjuvant approach, facilitating early tumor control while reducing delays in the administration of systemic treatment. Randomized studies, including RAPIDO and PRODIGE 23, have shown that total neoadjuvant therapy enhances disease-related outcomes, boosts treatment adherence, and improves pathological response rates in patients with high-risk rectal cancer, although the short course showed lower local control in RAPIDO but STELLAR did not show the same, indicating that short course can be used in certain scenarios [14,16,28]. The addition of immunotherapy to the short-course radiotherapy in locally advanced rectal cancer has shown a promising outcome. The TORCH/TORCH-C trial used short-course radiotherapy (5×5 Gy) followed by six cycles of toripalimab (PD-1 inhibitor) plus CAPOX chemotherapy with an overall clinical complete response rate of 58.1%. Ninety-five percent of the participants had microsatellite-stable and mismatch repair-proficient tumors. It proved that the "priming" effect of short-course radiation makes these "cold" tumors "hot" and sensitive to immunotherapy. Many patients were able to avoid surgery entirely through a "watch and wait" approach [29]. The UNION trial (phase II/III) also used short-course radiotherapy followed by camrelizumab (PD-1 inhibitor) and CAPOX chemotherapy. The phase II portion reported a pathological complete response rate of 48.1%. The three-year disease-free survival was 80.2%, and the overall survival was 93.3% [30]. All these trials suggested that combining short-course radiotherapy with immunochemotherapy might produce good results even in MSS rectal cancer. This supports our protocol of using pembrolizumab with CAPEOX and short-course radiotherapy.

This case supports the effectiveness of the multimodal strategy, despite the absence of a complete pathological response. The role of immunotherapy in this setting is of particular interest. While pembrolizumab is not standard in mismatch repair-proficient rectal cancer, its use may have contributed to improved systemic control. In addition, the immunogenic microenvironment of Epstein-Barr virus-associated nasopharyngeal carcinoma provides a strong rationale for checkpoint inhibition. Whether immunotherapy offers synergistic benefit in synchronous malignancies remains uncertain and warrants further investigation.

Retrospective analyses suggest that synchronous multiple primary malignancies are correlated with poorer outcomes compared to metachronous disease, primarily due to a greater tumor burden and increased complexity in therapeutic management [31]. Nonetheless, the current case illustrates that a robust and well-coordinated multimodal treatment approach, which includes systemic chemoimmunotherapy, radiotherapy, and surgical intervention, can lead to an excellent response and lead to curative outcome. In summary, this case underscores the vital importance of multidisciplinary collaboration in enhancing sequencing strategies, incorporating immunotherapy into multimodal treatment plans, and achieving a balance between systemic and local disease management. The continued collection of similar cases is crucial for the refinement of therapeutic strategies and the establishment of more standardized methods for the treatment of rare synchronous malignancies.

## Conclusions

This case underscores the viability of curative-intent management for selected patients diagnosed with synchronous nasopharyngeal carcinoma and rectal adenocarcinoma, achieved through a meticulously organized and sequential multidisciplinary approach. The combination of systemic chemoimmunotherapy, followed by an integrated short-course radiotherapy directed at the rectum and definitive concurrent chemoradiotherapy for the nasopharynx, and subsequent abdominoperineal resection illustrates the complex dual primary malignancy management. The optimal treatment can be fulfilled effectively without undermining oncologic principles if carefully planned multidisciplinary approaches have been followed.
